# Editorial: Infection-Related Rheumatic Diseases

**DOI:** 10.3389/fmed.2021.779773

**Published:** 2021-10-22

**Authors:** Konstantinos Thomas, Cassandra Calabrese, Dimitrios Vassilopoulos

**Affiliations:** ^1^4th Department of Internal Medicine, National and Kapodistrian University of Athens School of Medicine, Attikon University General Hospital, Chaidari, Greece; ^2^Department of Rheumatic and Immunologic Diseases, Orthopedic & Rheumatologic Institute, Cleveland Clinic, Cleveland, OH, United States; ^3^Department of Infectious Disease, Cleveland Clinic, Cleveland, OH, United States; ^4^Joint Rheumatology Program, Clinical Immunology-Rheumatology Unit, 2nd Department of Medicine and Laboratory, National and Kapodistrian University of Athens School of Medicine, Athens, Greece

**Keywords:** infections, rheumatic fever, vasculitis, Sjogren's syndrome, biologic therapies

The interplay between infections and rheumatic diseases has been a constantly evolving field for several reasons. First, a number of infections often manifest with primarily immunologic phenomena, as for example happens in hepatitis C virus-related cryoglobulinemic vasculitis or parvovirus or Chikungunya-associated arthritis. Second and more importantly, infections have been and continue to be among the most serious comorbidities in rheumatic patients treated with immunosuppressive or immune-modulating therapies. The latter issue has become more prominent in the last 20 years after the introduction of biologic and targeted synthetic therapies in daily clinical practice.

We present here a Research Topic collection of four articles entitled ***“Infection-Related Rheumatic Diseases,”*** hoping that it will provide a better understanding of this complex interaction between infection and rheumatic disorders.

In the first article of the collection, Calabrese et al. present their perspective on the contemporary landscape of infections after the launching of targeted (biologic and synthetic) therapies. They not only focus on infections associated with specific agents that were expected by preclinical research [i.e. tuberculosis and Tumor Necrosis Factor (TNF)-inhibitors], but also in severe infectious entities like progressive multifocal encephalopathy (PML) after natalizumab or rituximab that, although rare, need increased vigilance for their prevention and early detection. They also emphasize the significance of big data and post-approval surveillance for the documentation of potential links between novel therapies and specific infections. The authors finally suggest measures for early recognition of patients at risk for severe infectious complications, such as the use of prediction scores (1), and highlight the importance of vaccinations for the prevention of serious infections caused by respiratory and other pathogens (2).

In the second article by Ahn et al., the authors provide a nationwide estimation of tuberculosis incidence in patients with systemic necrotizing vasculitides (SNV) in South Korea. The issue of tuberculosis reactivation in patients treated with biologics, especially TNF-inhibitors, has been widely studied during the last 20 years and numerous scientific organizations have provided detailed guidelines for the effective screening and treatment of latent tuberculosis in rheumatic patients before biologic treatment initiation. However, the risk in patients with SNV treated mostly with conventional synthetic DMARDs and immunosuppressives has not been studied extensively. The authors reached to some interesting conclusions. First, they find a 6-time higher TB incidence in SNV compared to general population, both in men and women. Second, with the exception of eosinophinic granulomatosis with polyangiitis (EGPA), the finding was almost constant for other types of SNV. Third, the risk ratio was significantly higher during the first 3 months after diagnosis that aligns with the induction of remission regimens and the high glucocorticoid doses at the initiation of treatment in SNV. Despite some noteworthy limitations (high TB incidence country, dropout of more vulnerable patients, absence of association between infection and specific immunosuppressives), this is a valuable study for the better understanding of TB in this vulnerable population.

In a review article by Abdallah and Abu-Madi, the authors focus on the genetic background of rheumatic heart disease (RHD). Although rare in Western countries, the topic still is of great interest for a variety of reasons. The first is that the death toll in low- and middle-income countries still is worrisomely high (3), but moreover RHD disproportionately affects indigenous population even in high-income countries (i.e., Australia and New Zealand) (4). The second is that the migration of people from Africa and Asia to Europe will inevitably bring non-experienced physicians in these countries in front of this challenging diagnosis (5). It is more than clear that among the various manifestations of rheumatic fever, RHD is the one that carries the highest mortality and morbidity. The article includes a detailed graphic describing the complex immunologic pathways underlying RHD, recaps the recently revised Jones diagnostic criteria and more importantly delves into the RHD genetics presenting the associations between RHD and HLA and heavy immunoglobulin loci that were detected in the main genome-wide association studies (GWAS). The authors finally make a call for action to the direction of large-scale “omics” studies in RHD, using the recent advances in the study of autoimmune diseases.

Finally, there has been a speculation over long time about the potential involvement of viral infections in the pathogenesis of Sjogren's syndrome (SS). In this article collection, Hu et al. studied the effects of intratracheal administration of poly(I:C), a synthetic double stranded RNA mimicking viral infection, in an animal model of SS (SS-like NOD/ShiLtJ mouse). They found that poly(I:C) administration enhanced the inflammatory lesions in salivary glands, most likely through the action of interferon-induced signals and interleukin-33, a cytokine involved in Th1 signaling.

In conclusion, these articles show on the one hand how infections can participate in autoimmune processes and on the other hand, the potential of various immune-suppressive/modulating treatments used for the treatment of rheumatic diseases to increase the risk for common or opportunistic infections.

## Author Contributions

KT and CC conducted the manuscript. DV made the final amendments and approved the final version. All authors contributed to the article and approved the submitted version.

## Conflict of Interest

The authors declare that the research was conducted in the absence of any commercial or financial relationships that could be construed as a potential conflict of interest.

## Publisher's Note

All claims expressed in this article are solely those of the authors and do not necessarily represent those of their affiliated organizations, or those of the publisher, the editors and the reviewers. Any product that may be evaluated in this article, or claim that may be made by its manufacturer, is not guaranteed or endorsed by the publisher.
